# Disentangling the Roles of Approach, Activation and Valence in Instrumental and Pavlovian Responding

**DOI:** 10.1371/journal.pcbi.1002028

**Published:** 2011-04-21

**Authors:** Quentin J. M. Huys, Roshan Cools, Martin Gölzer, Eva Friedel, Andreas Heinz, Raymond J. Dolan, Peter Dayan

**Affiliations:** 1 Wellcome Trust Centre for Neuroimaging, University College London, London, United Kingdom; 2 Gatsby Computational Neuroscience Unit, University College London, London, United Kingdom; 3 Medical School, University College London, London, United Kingdom; 4 Donders Institute for Brain, Cognition and Behaviour, Centre for Cognitive Neuroimaging, Radboud University Nijmegen, Nijmegen, Netherlands; 5 Charité Universitätsmedizin Berlin, Campus Charité Mitte, Berlin, Germany; California Insitute of Technology, United States of America

## Abstract

Hard-wired, Pavlovian, responses elicited by predictions of rewards and punishments exert significant benevolent and malevolent influences over instrumentally-appropriate actions. These influences come in two main groups, defined along anatomical, pharmacological, behavioural and functional lines. Investigations of the influences have so far concentrated on the groups as a whole; here we take the critical step of looking inside each group, using a detailed reinforcement learning model to distinguish effects to do with value, specific actions, and general activation or inhibition. We show a high degree of sophistication in Pavlovian influences, with appetitive Pavlovian stimuli specifically promoting approach and inhibiting withdrawal, and aversive Pavlovian stimuli promoting withdrawal and inhibiting approach. These influences account for differences in the instrumental performance of approach and withdrawal behaviours. Finally, although losses are as informative as gains, we find that subjects neglect losses in their instrumental learning. Our findings argue for a view of the Pavlovian system as a constraint or prior, facilitating learning by alleviating computational costs that come with increased flexibility.

## Author Summary

Beautiful background music in a shop may well tempt us to buy something we neither need nor want. Valenced stimuli have broad and profound influences on ongoing choice behaviour. After replicating known findings whereby approach is enhanced by appetitive Pavlovian stimuli and inhibited by aversive ones, we extend this to withdrawal behaviours, but critically controlling for the valence of the withdrawal behaviours themselves. We find that even when withdrawal is appetitively motivated, it is still inhibited by appetitive Pavlovian stimuli and enhanced by aversive ones. This shows, for the first time, that the effect of background Pavlovian stimuli depends critically on the intrinsic valence of behaviours, and differs between approach and withdrawal.

## Introduction

The functional architecture of responding involves two fundamental components that are behaviourally [1] and computationally [2] separable: Pavlovian and instrumental. The instrumental component respects the stimulus-dependent contingency between responses and their outcomes (stimulus-response and action-outcome learning) [3]. By contrast, preparatory Pavlovian responses, chiefly involving approach and withdrawal, are elicited by the appetitive or aversive valence associated with predictive stimuli in a manner that is not dependent on the consequences of those responses [3]–[5].

The interactions between the two systems are most evident when automatically-elicited Pavlovian responses interfere with contingent instrumental responding [1], [6]–[9]. For instance, pigeons will strikingly continue to peck at a light predictive of food (a preparatory approach elicited by the appetitive prediction), even if the food is withheld every time they peck the light (the instrumental contingency) [10], [11]. Pavlovian interference likely contributes to many quirks of behaviour such as impulsivity [12], framing and [13], endowment effects [14] and many other “anomalies” [15], including neurological [16]–[19] and psychiatric diseases [20]–[26]. Further, puzzling facets of seemingly purely instrumental behaviour such as the difficulties in learning ‘go’ responses to avoid punishments; or ‘nogo’ to obtain rewards (unpublished data) and even the restrictions in associations evident in ‘evolutionarily preparedness’ [27], [28] might be traced to Pavlovian principles.

However, instrumental and Pavlovian systems share overlapping neural hardware. Their bidirectional interaction is characterised by two key triads: rewards are tied to approach and vigour; and punishments to withdrawal and behavioural inhibition. The neuromodulator dopamine (DA) responds predominantly to rewards [22], [29]–[31], induces behavioural activation and enhances approach [32]–[35]. Each aspect of this triad confounds the role of the phasic DA bursts in the flexible acquisition of instrumental values [36]–[42]. Serotonin appears to lie at the heart of the aversive triad, having been linked to punishments [43]–[45], behavioural inhibition and withdrawal [25], [32], [46]–[52], although dopamine acting via D2 receptors likely also plays a role in linking absence of rewards to nogo [17], [53], [54]. Signatures of both triads are also evident in neural circuits involved in response and choice. In the dorsal striatum, there are interdigitated pathways for ‘go’ and ‘nogo’, with the go pathways again linked positively to rewards via dopamine [16], [18], [55], [56]. The ventral striatum is primarily organized along an appetitive/aversive axis with direct links to approach and withdrawal behaviours [57], [58]. The aversive triad is also tightly linked to the dorsal raphé and the periaquaeductal gray [59], [60].

The main routes to the scientific investigation of these interactions consists of tasks in which Pavlovian stimuli are presented during ongoing instrumental tasks. However, these have as yet not explored the full set of interactions characterising the overlap between the two systems. Two critical confounds remain: The first confound concerns the precise nature of the effect of Pavlovian stimuli on instrumental behaviours. The instrumental behaviours studied have largely been appetitively motivated approach behaviours (in Pavlovian-Instrumental Transfer (PIT) and conditioned suppression tasks, [1], [6]–[8], [61]–[63]), and one instance of aversively motivated withdrawal behaviour [64]. The relative role of the appetitive-aversive motivation axis versus that of the approach-withdrawal axis is unknown. This in turn obscures the nature of the interaction: whether Pavlovian stimuli interact with the value of the instrumental behaviour, or by promoting specific responses [1], or even simply by modulating behavioural activation [5]. Second, the extent to which the separation of reward and punishment processing into opponent motivational structures applies to instrumental as well as Pavlovian learning is incompletely explored [1], [27], [28], [65].

**Figure 1:**
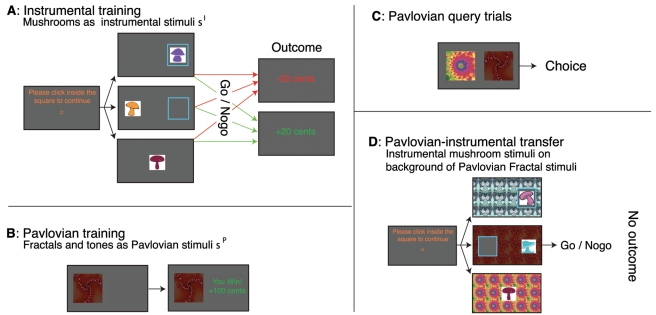
Task description. **A**: Instrumental training. To centre the cursor, subjects clicked in a central square. In approach trials (top), subjects chose whether to move the cursor towards the mushroom and click inside the blue frame onto the mushroom (go), or not do anything (nogo). In throwaway withdrawal trials (middle), they instead moved the cursor away from the mushroom and clicked in the empty blue frame (go) or did nothing (nogo). In release withdrawal trials (bottom), subjects were instructed to keep the button pressed after the initial click in the central square. The mushroom was then presented centrally, under the cursor. To throw away the mushroom, subjects released the button. Outcomes were presented immediately after go actions, or after 1.5 seconds. **B**: Pavlovian training. Subjects passively viewed stimuli and heard auditory tones, followed by wins and losses. **C**: On Pavlovian query trials, subjects chose between two Pavlovian stimuli. No outcomes were presented, but they were counted and added to the total presented at the end of the experiment. **D**: Pavlovian-instrumental transfer. Subjects responded to instrumental stimuli with Pavlovian stimuli tiling the background. No outcomes were presented, but subjects were instructed that their choices counted towards the final total. No explicit instructions about the contribution of Pavlovian stimuli towards the final total were given.

All these issues can simultaneously be addressed in a combined PIT and conditioned suppression task with both approach and withdrawal actions in which the overall motivational component of approach and withdrawal are matched (Figure 1 and Table 1). The task separates the contributions of approach and withdrawal by using two counterbalanced blocks, one involving approach go versus nogo, and the other withdrawal go versus nogo. The comparison between go and nogo controls for effects of behavioural activation or inhibition. In each block, subjects first underwent brief instrumental training (Figure 1A), learning from positive *and* negative feedback (monetary gains and losses of €0.20) whether to produce a go or a nogo response associated with sorting mushrooms. In the approach block (Figure 1A, top, all 46 subjects), go responses involved moving the cursor onto a mushroom (to collect it), while nogo involved doing nothing, thus not collecting the mushroom. To test for the effect of low-level motor variables, subjects performed one of two types of withdrawal actions. In “throwaway” (24 subjects, Figure 1A, middle), go involved moving the cursor physically away from the mushroom and clicking into an empty blue box; nogo involved doing nothing, and thus keeping the mushroom. Importantly, both approach to and withdrawal from the instrumental stimulus were orthogonal to any approach and withdrawal that might be directed at the Pavlovian background stimulus. In “release” (22 subjects, Figure 1A, bottom), the subjects had to start by pressing the mouse button. Go involved releasing the button to avoid collecting the mushroom; nogo involved continuing to press the button and thereby receiving the mushroom.

**Table 1:** Experimental layout.

Approach Block			
A1	Instrumental training (60 trials)		Probabilistic reinforcements^1^: 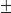 0.20 €
		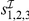 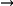 approach 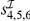 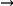 nogo	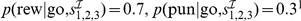 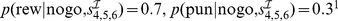
A2	Pavlovian training (60 trials)		Deterministic reinforcements
		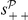 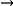 reward 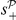 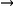 reward 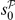 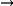 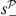 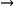 punishment 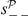 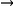 punishment	1 €0.10 €0−0.10 €−1 €
A3	PIT (100 trials)		No Reinforcements

Note the numerical subscripts on the instrumental stimuli 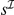 here refer to their identities, not to the time of presentation.

1 For subject with deterministic instrumental reinforcements, the outcome probabilities were 1 and 0 instead of 0.7 and 0.3, respectively.

In order to orthogonalise the approach-withdrawal and appetitive-aversive axes, the *learned* instrumental values in approach and withdrawal blocks needed to be matched. To achieve this, both go and nogo responses were, if correct, rewarded. Additionally, to avoid the confound of activation, in each block (i.e. in both approach and withdrawal blocks) the go action was designated as the correct response to half the instrumental stimuli, and the nogo action to the other half (see Table 1). Incorrect responses had opposite outcome contingencies to correct responses, yielding more punishments than rewards. This ensured that go, nogo, approach and withdrawal overall had the same learned association with rewards and punishments. We tested both deterministic and probabilistic outcomes but found no differences.

In the second part of each block, subjects passively viewed unrelated, fractal, stimuli paired with separate auditory tones (Figure 1B). Each compound Pavlovian stimulus 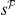 was deterministically associated with a monetary gain or loss, i.e. its Pavlovian value 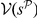 was equal to that monetary outcome. Every fifth trial in the Pavlovian block was a query trial (Figure 1C), in which subjects chose the better of two fractal visual stimuli without being informed about the outcome. Finally, in the PIT stage, the instrumental stimuli were presented on a background of fractal Pavlovian stimuli together with the auditory tones, and again without outcome information.

Our task addressed the key confounds described above. With respect to the triads, we found that the Pavlovian influence is action specific: appetitive Pavlovian cues boosted go approach responses and suppressed withdrawal go responses; aversive Pavlovian cues did the opposite. Additionally, subjects were substantially biased against withdrawal, but we found no evidence that the instrumental learning component itself differed between the approach and withdrawal condition.

## Results

The key results in this paper concern the interaction of valued Pavlovian stimuli on instrumental choices. We first present a direct analysis of the choice data and reaction times. We then provide a detailed modelling analysis of the data, employing a stringent form of group-level model selection that assesses each model's parsimony by weighing its ability to fit the data against its complexity. The models quantify Pavlovian values 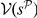, which are the expectations of a gain or loss given Pavlovian stimulus 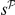, and instrumental choice values 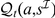, which are the time-varying expectations of a reward given a response 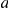 to an instrumental stimulus 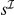. The structure of the most parsimonious model implies the influences and interactions that were significant (for instance ruling in a bias against active withdrawal, but ruling out any difference between the instrumental learning rates associated with approach and withdrawal); the values of the parameters in this model indicate the nature of those influences and interactions.

### Model-free analyses

**Figure 2:**
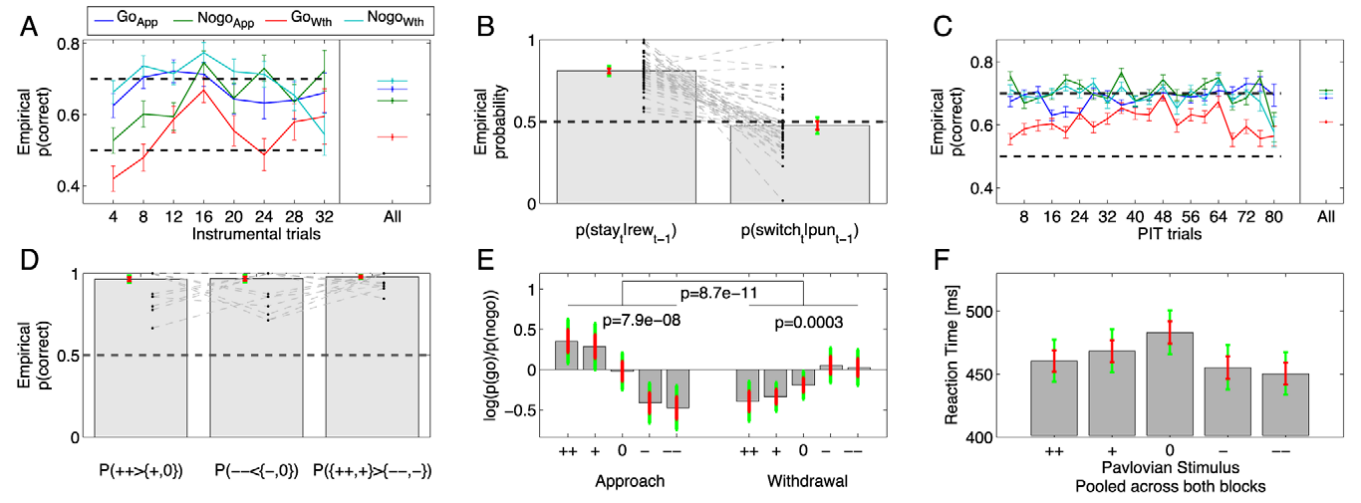
Raw choice probabilities. **A&C**: Average probability (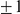 standard error) of choosing the more rewarded (“correct”) action in the instrumental (A) and PIT (C) parts. Average performance was above chance in all cases, but worse when withdrawal go was the more rewarded action (red). There was no extinction during the PIT block. Each point is the average across subjects and across four trials. **B**: The bars show mean overall probability of repeating an action in the instrumental part given that it was last rewarded in the presence of the current stimulus, or the probability of switching given a previous punishment. Punishments do not lead to reliable switching. **D**: Choice probabilities in the Pavlovian forced choice query trials. Most subjects were close to perfect. The grey bars show the probabilities of *left*: choosing a very good stimulus (++) over a good (+) or neutral (0) stimulus; *middle*: choosing a bad (−) or neutral (0) stimulus over a very bad (--) stimulus; *right*: choosing a positive (++ or +) stimulus over a negative one (-- or -). Subjects that performed submaximally in the appetitive Pavlovian domain did not necessarily have lower reward sensitivities in the instrumental task, and vice versa for aversive Pavlovian stimuli and punishment sensitivity. **E**: PIT effects. The left part shows the approach PIT block, the right part the withdrawal PIT block. Each bar shows the log ratio of the choice probability (go/nogo) in the presence of one of the five Pavlovian stimuli. There was a significant effect of Pavlovian stimulus valence in each block. In addition, there was a significant block
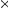
Pavlovian stimulus valence interaction. Grey bars are means 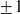 standard error (red) and 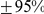 confidence intervals (green). **F**: Reaction times, pooled data for both PIT blocks. The bigger the absolute valence of the Pavlovian stimulus, the shorter the reaction time.

There was no difference between the results for probabilistic and deterministic feedback, and we therefore present the combined data. Analysis of the components of the experiment indicate robust, yet moderate, instrumental conditioning that was stable during the PIT period, combined with highly robust Pavlovian conditioning. Figure 2A shows the instrumental probability of choosing the more rewarded (“correct”) stimulus over time. Subjects rapidly came to prefer the more rewarded action. Preference was weaker for go withdrawal, against which there was a consistent bias. We intended the instrumental preference to be weak to avoid ceiling effects when assessing PIT.

**Figure 3:**
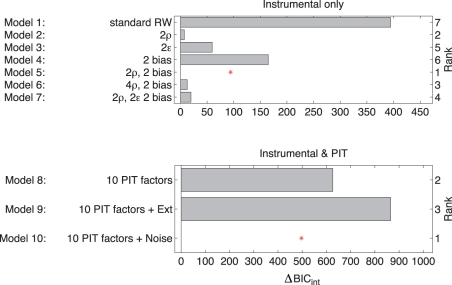
Model comparison. Each bar shows the differential 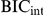 score relative to the model with the lowest 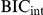 score (log 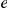 scale). Note that these 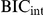 scores are for the group as a whole. **Top**: Models 1–7 were fitted to the instrumental data only. Model 1 was a standard Rescorla-Wagner type model which forced rewards and punishments to be equally informative. It assumed equally fast learning about rewards and punishments, and no biases. Inclusion of either separate reward and punishment sensitivities (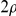, Model 2) or separate biases in the approach and withdrawal blocks (Model 4) improved the fit. Separate learning rates for rewards and punishments (Model 3) did not improve the fit as much as separate reward and punishment sensitivities (Model 2). The best model (5) included a separate go bias in the approach and withdrawal blocks, and separate reward and punishment learning rates. Models that additionally allowed separate reinforcement sensitivities (Model 6), or separate learning rates (Model 7) in the approach and withdrawal blocks failed to improve the fit. **Bottom**: Comparison of models on both instrumental and PIT choice data jointly. Models 8–10 used the instrumental component of Model 5. Models 8–10 included ten Pavlovian factors, capturing the effect of each of the five Pavlovian stimuli in each of the two blocks. Model 9 allowed for extinction by including an exponential decay of the instrumental values during the PIT part of the task. Model 10 included random generalisation noise and provided the best fit.

Subjects also exhibited predictable variability on a shorter time-scale: Figure 2B shows the immediate consequences of rewards and punishments on subsequent behaviour. It is notable that punishments did not reduce the repeat probability below chance level (mean 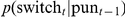 is not 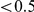, one-tailed t-test 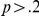). The same was found when analysing go and nogo choices separately: in both cases, 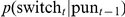 was not significantly different from 0.5 (both 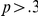, two-tailed t-test), and was significantly smaller than 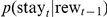 (both 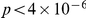, paired t-test). Whether this really does represent an insensitivity to punishments depends, however, on the average stay probability, and on how this average stay probability is related to past reinforcements. Subjects were instructed that the outcomes of responses in the PIT block would be counted as in the instrumental block. Figure 2C shows that this led to stable maintenance of the instrumental response tendencies throughout the PIT block. Figure 2D shows that all but one (excluded) subject showed extremely good performance on the Pavlovian query trials interleaved with the Pavlovian training (mean correct 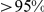).

Given the success of instrumental and Pavlovian training, we next analysed the raw effect of Pavlovian stimuli on approach and withdrawal choices. Figure 2E shows a highly significant interaction between block and Pavlovian stimulus valence. Relative to neutral stimuli, positive Pavlovian stimuli enhanced approach and inhibited withdrawal go over nogo. Conversely, negative Pavlovian stimuli enhanced withdrawal and inhibited approach go over nogo. A similar analysis looking at the probability of responding incorrectly (outside the blue box) showed no effect of the Pavlovian stimuli in either approach or withdrawal condition and no interaction (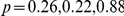 respectively, ANOVA), suggesting that these results were not due to response competition. Note that the withdrawal go probabilities were lower than the approach ones, again reflecting the overall bias against go withdrawal.

Average reaction times for go approach and go withdrawal actions did not differ (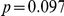, 2-tailed t-test). Against our expectations, Pavlovian stimuli of both positive and negative valence shortened reaction times in a parametric manner relative to neutral Pavlovian stimuli (Figure 2F, p = 0.0310, ANOVA), although this effect was not present in either block separately (p = 0.5502 and p = 0.0781 respectively, ANOVA).

### Model-based analyses

The size of the PIT effect may have been affected by the extent of instrumental learning (and thus the actual learned action values), by response biases, and by generalization from the instrumental to the PIT stage. In addition, there may have been differences in the instrumental learning of approach and withdrawal actions (Figure 2A). We decomposed and analysed all such factors using a detailed reinforcement learning model. This contained explicit parameters capturing all the instrumental and Pavlovian effects in the task, and was fit to the choice data of all subjects. We used group-level Bayesian model comparison [66] to choose amongst a variety of model formulations (reporting 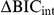 scores relative to the final model), and ensured that inference yielded correct parameter estimates when run on surrogate data generated from the assumed underlying decision process.

### Instrumental learning

The final model included 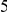 parameters associated directly with the instrumental requirements of the task. These comprise one learning rate 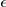; two parameters 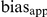 and 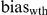 representing the bias towards go in the approach and withdrawal blocks; and two separate free parameters 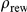 and 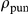, representing the effective strengths of rewards and punishments.

**Figure 4:**
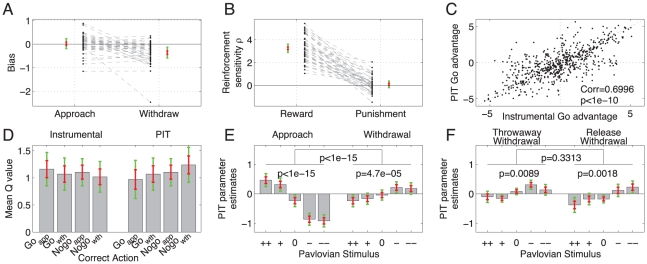
Instrumental model parameters. **A**: Go biases for the approach and withdrawal condition in the full experiment. Subjects were only biased against go, compared to nogo, in the withdrawal block. **B**: Reward and punishment sensitivity. Subjects were significantly more sensitive to rewards than punishments. **C**: Generalization noise. Effective 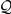 value differences between go and nogo actions for all stimuli and subjects, at the end of instrumental learning and during the PIT block. Generalization seemed noisier when action preferences were weaker. **D**: Mean 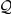 values of ‘correct’ (i.e. more frequently rewarded) actions. There was no difference, and all correct actions had *positive* expectations on average. **E**: PIT parameter estimates, correcting for instrumental learning, response biases and generalization noise. Positive Pavlovian stimuli enhanced approach go actions and inhibited withdrawal go, while negative Pavlovian stimuli inhibited approach go actions and enhanced withdrawal go actions. The interaction was highly significant, as were the two linear main effects. **F**: There was no difference between the effect of Pavlovian stimuli on throwaway versus release go actions (all 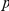 values in E and F are ANOVA). Throughout, grey bars are prior means with estimates of standard error (red) and 95% confidence interval (green). Black dots show individual data points, and individual subjects' parameters are connected by a dashed grey line in A and B.

At a group level, subjects were biased against active withdrawal, but showed no bias for or against approach (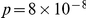 and 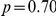 respectively, two-tailed t-test), the difference being significant (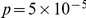, ANOVA, Figure 4A). Withdrawal biases in the release and throw away experimental subgroups did not differ (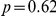, ANOVA), controlling for motor effects. The withdrawal bias accounts for the lower performance on go withdrawal in Figure 2A.

**Figure 5:**
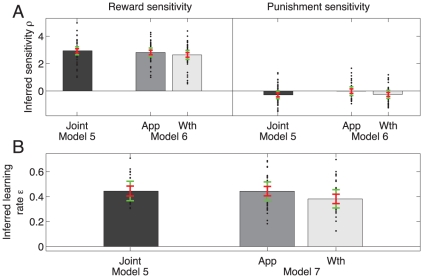
Reward sensitivities and learning rates in instrumental approach and withdrawal blocks do not differ. **A**: The dark bars show the reward (left) and punishment (right) sensitivities in Model 5, which collapses across approach and withdrawal conditions. The grey and light grey bars show the sensitivities when fit separately for approach and withdrawal blocks (Model 6). There is no difference between blocks; and the joint parameter differs from neither (all pairwise comparisons 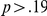). **B**: Dark bar shows learning rate collapsed across both conditions in Model 5. Grey and light grey bars show learning rates when fit separately for approach and withdrawal condition. Again, no pairwise difference is significant (all 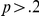). Throughout, black dots show individual data; bars show prior means and red and green error bars 1 estimated standard error and 95% confidence interval, respectively.

One concern is that differences in the biases might have masked differences in learning (i.e. the reward sensitivities) in the approach and withdrawal conditions. We tested this by allowing for separate reward and punishment sensitivities in the two conditions (Model 6) or separate learning rates (Model 7). The use of these extra parameters was structurally rejected by the model selection process (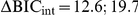 respectively for the purely instrumental trials); and the freedom to choose different parameter values in these conditions was duly not used (Figure 5). The absence of any difference in the *learning* parameters for approach and withdrawal suggests that the instrumental system treated approach and withdrawal entirely equally. We will see below that this was not true for the Pavlovian system.

Although, by design, rewards and punishments were equally informative, subjects chose to rely more on rewards than punishments (Figure 4B). Rewards had a stronger effect than punishments both at a group level and for all individual subjects, the difference being significant (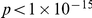, ANOVA). Indeed, the average punishment sensitivity was not distinguishable from zero (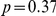, two-tailed t-test). This remained true when we separately tested subjects who were given deterministic (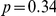, two-tailed t-test) and probabilistic (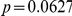, two-tailed t-test) feedback. Supplementary analyses (Text S1) excluded two further explanations for the punishment insensitivity: first, that it is due to choice perseverance (Figure S1 Text S1); and second that it is due to an emerging maximisation behaviour (Figure S2 in Text S1). Thus, it appears that the pattern seen in Figure 2B is indeed due to a differential sensitivity to rewards and punishments.

### Generalization: Extinction versus noise

We next analysed the generalization of instrumental 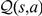 values from the instrumental to the PIT blocks. Generalization could be imperfect in two ways - the starting 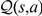 values in the PIT block could differ from the ending 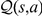 values in the preceding instrumental block, and the 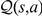 values could then decay over time or trials during the PIT block given the lack of information about the outcomes. We constructed models including such effects, and tested whether their excess complexity was outweighed by their fit to the data.

As expected from the stable raw probabilities of choosing the correct (i.e., more rewarded) option (Figure 2C), a model in which the instrumental 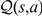 values decayed exponentially over time during the PIT block (mimicking extinction) did not provide a good account of the data (Model 9, compared to Model 10 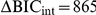).

Rather, the final model allowed for the addition of random generalization noise to each 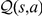. These factors were drawn independently from the same normal distribution for all stimulus-action pairs, and the mean and variance of this distribution were both inferred without constraints (see Methods). Figure 4C visualizes the resulting changes; each dot represents the preference for the go action (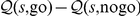) for all subjects and all stimuli. The abscissa shows this at the end of the instrumental stage, the ordinate after addition of the noise for the PIT stage. Importantly, there was no systematic difference in mean correct action values either in the instrumental or PIT stage (Figure 4D).

### Pavlovian-Instrumental transfer

We were mainly interested in the effect of the Pavlovian values on instrumental performance. We therefore fitted 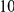 unconstrained parameters to separately capture the influence of each of the five Pavlovian stimuli on instrumental go actions in both the approach and withdrawal condition.

All models accounted for performance in the PIT part by adding up instrumental and Pavlovian influences prior to taking a softmax [67], [68]. This amounts to treating instrumental and the Pavlovian controllers as separate experts, each of which ‘voted’ for its preferred action. The model captured in detail, and thereby controlled for, variability in instrumental learning and generalization. The final model predicted the choices of every individual subject better than chance (binomial probability, 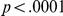 for every subject, overall predictive probability 0.7544). The maximum a posteriori (MAP) estimates of this model's parameters painted a picture very similar to that seen in the raw data.

Figure 4E shows the parameters of the model related to the influence of each Pavlovian stimulus. The pattern mirrored that seen in the raw data: there are highly significant, and opposite, effects in the approach and withdrawal blocks, with appetitive stimuli (++ and +) promoting approach but inhibiting withdrawal; and aversive stimuli (-- and -) promoting withdrawal but inhibiting approach. At a single subject level, the effect in the approach block was seen in 45/46 subjects (98%), while it was seen in 30 subjects (65%) in the withdrawal block.

Since there was no difference in the learned value of go or nogo actions in either approach or withdrawal blocks, and in either the instrumental learning or the PIT stages (Figure 4D), any PIT effects are unlikely to be due to a preferential association of a Pavlovian stimulus with the learned value of an action. Rather, they reflect the approach or a withdrawal nature of the action.

We included two separate groups of subjects who either performed a throwaway withdrawal action, or a release withdrawal action. This was both to test the contribution of an approach/withdrawal component aimed at the Pavlovian stimuli tiling the background, and in recognition of the sophistication of defensive reactions [27]. Figure 4F shows that Pavlovian stimulus value had a significant, linear effect on both withdrawal action types, and that this overall linear effect did not differ between the two action types. At an individual level, linear correlations were positive for 16 (72%) and 14 (58%) subject in the release and throwaway condition, respectively.

### Psychometric measures

No psychometric measure of anxiety or depression correlated with any of the parameters in the main model.

## Discussion

Our task was designed to look inside the triads of valence, behavioural activation and inhibition, and specific actions associated with Pavlovian influences. This issue has been incompletely explored in the past. Either these triads as a whole have been investigated: aversive actions allowed avoidance of, or escape from, a negative reinforcer; appetitive actions, the acquisition of a reward [6], [8], [64], or, as in negative automaintenance [10], the relevant Pavlovian contingencies have been tightly embedded in the instrumental task. Here, we found that Pavlovian influences distinguished approach from withdrawal when carefully controlling for activation, for appetitive versus aversive instrumental motivation, and for details of the motor execution. Thus, for instance, a Pavlovian stimulus predicting reward had opposite effects on two different instrumental actions (approach and withdrawal) even though both those actions were themselves equally motivated by the acquisition of reward.

Approach and avoidance were defined in two parallel ways: by the cognitive label for the action (‘throw away’, ‘collect’) and by the relation to the stimulus (moving the mouse/finger towards or away from the stimulus). Our task did not set out to distinguish these two contributions (cognitive and motor), and we also did not attempt to quantify subjects' explicit insight into their strategies.

However, both possibilities are important. At a cognitive level, subjects should neglect the Pavlovian stimuli: by design, they are not informative about the instrumental task. Upon entering the PIT stage, subjects were also explicitly instructed to continue doing the instrumental task as before. If despite these facts subjects were cognitively swayed to include the irrelevant backgrounds in their goal-directed decision process, then our finding show that Pavlovian contingencies extend even into cognitive choices. This is of course consonant with a large number of behavioural irregularities in human decision making [12]–[15].

The motor aspects are equally interesting since they suggest a fine level of detail in the architecture of Pavlovian influences. There is quite some evidence for this; for instance, Pavlovian CRs are known to be highly adaptive to the details of the CS (for instance evoking a grooming conditioned response to a rat which functions as a food CS, rather than a gnawing CR [69]) and to the nature of the US [70]. In humans, a plexiglass positioned between subjects and an appetitive US abolishes an increased willingness to pay [71].

The performance on the purely instrumental portion of the task was also revealing. We observed a difference in the instrumental performance of approach and withdrawal action; and this came (unlike in previous tasks) after controlling for the motivational difference between approach and avoidance. Our model-based analysis revealed that the difference was not due to a difference in learning (i.e. a difference in the instrumental parameters relating reinforcements to performance), but due to a static bias against performing a withdrawal go action. Of course, like all other tasks, our instrumental task also had embedded Pavlovian contingencies, and, indeed, a Pavlovian suppression of active withdrawal by the overall appetitive framing of the task (subjects on average chose the correct, rewarded, action more often) could mirror what we saw in the PIT stage of the task. Alternatively, this could be the result of subjects' experiences upon entering an experimental situation in which they are given a computer mouse. We have interpreted such as bias in terms of evolutionary preparedness or programming [2], [9], [24], [50], [72]. That is, the flexibility of the arbitrary outcome-contingent mappings of instrumental control comes at the price of the experience necessary for it to be specified. Pavlovian priors substitute inflexible hard-wired choices that are immediately available for this flexible instrumental adaptativity with its potentially substantial sample complexity (i.e. the potential need for extended experience). Related biases are widely known: dogs will happily learn to run, but not to yawn, for food; teaching a rat to escape is easier than teaching it to avoid the shock [3], [27], [28]; humans perform active go responses slower if instructions are in terms of aversive feedback [51] or if they are followed by aversive information [73]. Finally, in humans, an instructed joystick approach response to a happy face is quicker than a withdrawal response, depending on the cognitive/affective label in a manner similar to our own findings here [74].

Alternative interpretations of the response bias include endowment effects [14], whereby an over-valuation of items notionally in one's possession makes one reluctant to give them up. This is unlikely because such a bias should be present across all instrumental stimuli, i.e. across both stimuli for which a go and a no-go is the more rewarded action (Figure 4). Another possibility is a frame dependence [13]—since we compared go with nogo rather than two alternative go actions against each other. The negative frame associated with sorting to remove bad mushrooms could have inhibited go actions.

### Neurobiology

One of the central motivations for our investigation was the observation that the neural substrate does not respect the logical independence of reward/punishment and approach/withdrawal. Rather, as we have discussed, these are tied together, via the structure of the striatum and also specific neuromodulators.

While the neural basis for the promotion of approach responses by appetitive stimuli is known to involve both amygdala and striatum [62], [63], [75], the neural bases for the effects of aversive Pavlovian stimuli are less clear. There are no data on withdrawal responses per se, i.e. with positive expectations. Nevertheless, animal models, genetic studies and pharmacological manipulations suggest that serotonin plays a crucial role in the inhibition of active behaviours by aversive expectations [25], [47], [48], [50], [73], [76]–[78]. In humans, there is evidence for the serotonergic mediation of the inhibition of active approach by aversive predictions [51], and of approach responses to stimuli that are predictive of negative reinforcement [73]. It should be noted, though, that, acting via the indirect path and D2 receptors, dopamine itself has also been suggested to be important in mediating ‘nogo’ behaviour due to punishments [18], [53], [79].

Aversive Pavlovian stimuli can also potentiate behaviour [1], [64], [80], [81], with both serotonin and dopamine involved. Dopamine may have a dominant influence in this: it is both known to be released, and influential, in some aversive settings [82]–[85] and has a more evident relationship to vigour [33], [34]. This observation has led to a re-interpretation of previous notions [43] of the opponency between dopamine and serotonin, putting an axis spanning invigoration and inhibition together with spanning reward and punishment [52].

Thus, the literature suggests three predictions for genetic correlates of the Pavlovian influences we observe. When considering these, the caveats concerning the interaction of genetic variation with psychopathology (e.g. anxiety or depression), and with development need to be kept in mind. Nevertheless, the conditioned suppression effect of aversive Pavlovian stimuli on approach should be enhanced by D2 receptors, and hence be positively related to D2 striatal receptor density thought to be modulated by C975T (rs6277; [17]). Second, conditioned suppression should be increased in subjects with higher serotonin levels, i.e. as might be the case with the less efficient (s) allelic variation of the serotonin reuptake transporter (5HTTLPR SLC6A4 [86]). Third, given dopamine's established positive correlation with approach and PIT [87], [88], we expect genetic polymorphisms that boost DA levels, such as the SLC6A3 polymorphism of the dopamine transporter [89], to increase the impact of appetitive Pavlovian stimuli on approach. A similar effect may be expected from DARPP-32, although its closer relationship to synaptic plasticity would also suggest effects on instrumental learning [90]–[92].

### Instrumental punishment insensitivity

Although the learning parameters associated with instrumental approach and withdrawal did not differ, the impact of rewards and punishments on the acquisition of responding was highly asymmetric. In general, subjects neglected punishments, whilst maintaining a fixed sensitivity to reward. This was gratuitous as, in our setting, rewards and punishments were equally informative. It is, however, the case that the optimal strategy can be arrived at by concentrating on either.

Subjects were not globally insensitive to punishments, as their choice behaviour in the Pavlovian learning was highly accurate both for rewards and punishments. Furthermore, it should be emphasized that ascribing punishments a value of zero outcome would still effectively behave as a punishment because a zero outcome is well below the average expectation of correct actions (Figure 4D) and as such would reduce the tendency to emit the action that caused it. The asymmetry has been noted before. Others have fitted models with separate learning rates for rewards and punishments and reported significantly slower learning rates for punishments than rewards [93], [94]. In some restricted regimes, learning rates and inverse temperature parameters can trade off, and we explicitly tested both types of models to address this.

One potential confound is the emergence of determinism. Subject were instructed to perform choices relative to mushrooms. Real world mushrooms are either edible or poisonous, and this dichotomy may have predisposed subjects towards a deterministic, rather than a matching, strategy. (For instance, subjects may have chosen responses based on a classification of the mushrooms into ‘good’ and ‘bad’ ones, rather than on the particular value of a response for a mushroom.) Indeed, in RL settings it is typically optimal to start with a low, exploratory, sensitivity to outcomes, but to increase this over time to encourage exploitation, culminating in a deterministic strategy [2]. However, subjects did not behave deterministically at any point (Figure 2A) and supplementary analyses showed that the time-varying pattern of reinforcement sensitivities this would predict is not observed in the data (Text S1). A further potential confound is the average stay probability. If this were precisely half-way between the stay probabilities after rewards and punishments in Figure 2B, then rewards and punishments would have the same effect relative to the baseline, and hence arguably be equally informative. However, this argument would neglect the fact that the mean stay probability itself must be a function of the reinforcement history; and that this must be included in making inferences about the reinforcement sensitivity.

We have previously made the argument on theoretical grounds that part of the asymmetry observed in appetitive and aversive systems might be due to the inherent difference in how informative rewards and punishments are processed, enshrined again in the architecture of the striatum and neuromodulation [50]. Rewards tell us what to do; punishments tell us what not to do. The former is more informative in naturalistic settings where many options are available but only few are good. The fact that subjects gratuitously rely on rewards rather than on punishments in the present setting may reflect an implicit appreciation of this fact, although our findings are certainly in no way conclusive evidence. Interestingly, it is known that stronger optimality results can be shown for a stochastic learning automata rule called linear reward-inaction, which does not change propensities in the light of punishments but only rewards ([95], [96]; also known as a benevolent automaton [97]), than for a rule that changes propensities for both.

### Modelling

The computational model served several central roles. First, it encapsulated the manifold aspects of behaviour and learning *jointly*, thereby controlling for them: the bias against withdrawals is not a due to a difference in learning; and variations in learning or generalization do not account for the PIT effects we saw. Secondly, its close fit to the behaviour argues that the PIT effects can be accounted for by a simple superposition of an instrumental and a Pavlovian controller: the action propensities due to both controllers were simply multiplied (as additive factors in an exponential), rather than being allowed to interact in more complex ways.

The simplicity of this interaction eschews questions about peripheral versus central response competition, whether appetitive and aversive systems compete centrally [7], and whether Pavlovian learning is involved in instrumental learning [1]. It takes the view of multiple, separate controllers contributing in parallel [98], and weighting the ultimate choice by the reward expected from that choice. One alternative would be to weigh contributions by different controllers according to their certainty [99], although it is unclear how to compute the Pavlovian controller's certainty.

### Limitations

There are various pressing directions for future studies. First, despite the role the architecture of decision-making has played in the argument, our work does not directly address the neural mechanisms concerned. These could be examined using imaging and pharmacological manipulations.

Second, our task was not designed to distinguish between outcome-specific and general mechanisms [63], [75] as we relied on one, monetary, outcome throughout. Studying different outcomes is important, given evidence for partly parallel pathways through different nuclei of the amygdala and different targets in the nucleus accumbens [100], [101].

Third, we are missing one crucial further orthogonalization to do with the overall framing of the instrumental task. It is important to consider the case in which subjects can at best avoid losing money by doing the correct action [51]. We would expect punishment to maintain its instrumental force in this case; but there could also be a systematic difference in the nature of the Pavlovian influences.

### Conclusion

Pavlovian responses are believed to be hard-wired to reflect evolutionarily appropriate attitudes to predictions, being highly adaptive and sensitive to environmental structures [102]. Here, we showed that Pavlovian influences on instrumental behaviour depend on the intrinsic affective label of an action, independent of its learned reward expectation.

It has long been known that prepared or compatible [27], [69] behaviours are easier targets for instrumental conditioning. These intrinsic biases, or priors, may serve a crucial function both by reducing the need for collecting data (i.e. sample complexity) about the effects of actions, and by reducing the need for executing complex processing necessary to work out optimal actions (i.e. computational complexity). Both of these can be expensive or dangerous, particularly in an aversive context. Our findings sharpen the understanding of the relative contribution of Pavlovian and instrumental contingencies in general tasks. We showed clearly that the interaction of Pavlovian and instrumental behaviours is organized along the lines of appetitive and aversive motivational systems, and that a critical contributor to this is the affective nature of actions.

## Methods

### Subjects and procedure

54 healthy subjects of central European origin were recruited from the Berlin area. Subjects were screened for a personal history of neurological, endocrine, cardiac and psychiatric disorders (SCID-I screening questionnaire), and for use of drugs and psychotropic medication in the past 6 months. Subjects received performance-dependent compensation (5–32 Euro) for participation. Three subjects did not meet inclusion criteria and one subject did not complete the task; the data for three further subjects were lost due to a programming error. One further subject was excluded from the analysis because the instrumental task was not satisfactorily performed. The 46 remaining subjects were 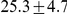 years old. 59% were female (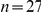). The study was approved by the local Ethics Committee and was in accord with the Declaration of Helsinki 2008. Subjects were given detailed information and gave written consent. They were seated comfortably at a table in front of a laptop with headphones and used a mouse with their dominant hand to indicate their choices. The amount earned was indicated by the computer, and the sum paid in cash at the end of the session. The computer task was followed by completion of self-rating scales.

### Task description

The task was written using Matlab and Psychtoolbox (http://psychtoolbox.org). It consisted of one approach and one withdrawal block separated by a 2 minute break. Each block was in turn divided into a instrumental training, a Pavlovian training and a PIT part. Table 1 illustrates this.

#### Instrumental training

The instrumental task was framed in terms of a mushroom collecting and sorting task. Instrumental stimuli were generic, coloured mushroom shapes. Trials started when subjects clicked in a central square (Figure 1A). In the approach block, instrumental stimuli 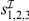 and 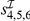 (with subscripts indicating the *identity* of stimuli, not the time of presentation) were then presented to one side, surrounded by a blue frame (Figure 1A, middle column, top). Subjects indicated that they wanted to collect the mushroom by moving the cursor onto the mushroom and clicking on it (approach go). They could also decide not to collect the mushroom by doing nothing for 1.5 seconds (approach nogo). At the end of each trial (after a click for go trials or after 1.5 s for nogo trials respectively), the stimulus disappeared and the outcome was shown in the middle of the screen (Figure 1A). In the withdrawal blocks, instrumental stimuli 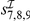 and 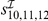 were presented. Subjects chose whether to throw away mushrooms (withdrawal go) or do nothing (withdrawal nogo). Two different withdrawal go actions were tested. The ‘throwaway’ group (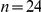) had to click in a blue frame located on the opposite side of the stimulus (see Figure 1A, middle column, middle). The ‘release’ (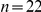) group was instructed to press *and hold* the mouse button after clicking in the central square to begin the trial. The mushroom was then presented underneath the cursor (Figure 1A, middle column, bottom), and they could throw away a mushroom by releasing the button (withdrawal go) or not throw away the mushroom by not releasing (withdrawal nogo) until 1.5 seconds had elapsed. Each block contained three “good” (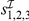 and 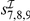) and three “bad” (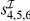 and 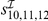) mushrooms, randomly selected from the pool of 12 stimuli. Subjects were given explicit reinforcing feedback after every choice (‘Correct, +20 cents’ or ‘Wrong. −20 cents’), either deterministically (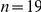) or probabilistically (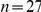), but were not told which mushrooms were good or bad. Correct trials were those on which subjects threw away a bad or kept a good mushroom, and those on which they collected a good or refrained from collecting a bad mushroom. Importantly, this means that correct go actions of both types (approach (‘collect’) and withdraw (‘throw away’)) were followed by both rewards and punishments. Thus, the reinforcement expectancies of correct approach and withdrawal actions were equal and positive on average. Similarly, incorrect actions of both types were also followed by rewards and punishments, but more by the latter than the former. To ensure replicability across experimental designs, four experimental configurations were included, crossing deterministic/probabilistic instrumental feedback and the two withdrawal action types (‘throw away’ or ‘release’). These manipulations are beyond the mathematical model described below, and thus should not affect our findings. We present both data for all subjects and, testing internal consistency, across the four groups. 10 subjects were in the deterministic throwaway group, 9 in the deterministic release, 14 in the probabilistic throwaway and 13 in the probabilistic release group. One-way ANOVA comparisons of MAP parameter estimates from the most parsimonious model (Model 10; see below) for deterministic and probabilistic feedback did not reveal any significant differences.

#### Pavlovian training

Five compound Pavlovian stimuli consisting of a fractal visual stimulus (Figure 1B) and a tone were classically conditioned. Each stimulus was presented 20 times and deterministically followed, 1 second later, by the associated outcome. Outcome presentation lasted 1.5 seconds. Outcomes for the best (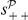), good (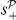), neutral (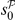), bad (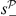) and worst (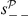) stimuli were, respectively, gains of 100 cents, 10 cents, zero, and losses of 10 and 100 cents. To ensure that subjects paid attention, every fifth trial was a query trial in which subjects had to choose between two Pavlovian stimuli (Figure 1C). No feedback was given in these trials, but subjects were instructed that the choices would contribute to their compensation.

#### Pavlovian-Instrumental transfer

In the final part of each block, the instrumental task was presented in extinction and on the background of Pavlovian stimuli (Figure 1D). Subjects were instructed to continue doing the instrumental task; that choices were still earning them the same outcomes and were being counted, but that they would not be told about the outcomes. Note, importantly, that the Pavlovian stimulus was presented over the entire background, and as such could not by itself modulate the directionality of actions.

#### Psychometric measurements

After completing the tasks, subjects completed self-rating scales (Beck Depression Inventory II (BDI), Beck Anxiety Inventory (BAI), State-Trait Anxiety Inventory STAI [103]–[105]), followed by the administration of clinician rated scales (Montgomery-Ashberg Depression Rating Scale (MADRS), Hamilton Depression Scale (HamD), Structured Interview for the Hamilton Anxiety Scale (SIGHA) and Clinical Global Impression (CGI) [106]–[108]).

### Models

We modified a standard reinforcement learning model to capture the behavioural choices in the experiment. We first describe the main model, and then the alternative control models. Considering first the instrumental part, let 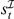 be the instrumental stimulus (out of up to 12; i.e. the subscript 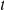 now designates *time* rather than identity as in Table 1) presented at trial 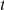, and 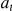 the action (choice) on that trial. An action can be one of four types: go withdrawal and nogo withdrawal in the withdrawal block, and go approach and nogo approach in the approach block. Let also 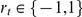 be the reinforcement obtained, either 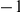 for a punishment, or 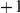 for a reward. We write the probability of action 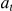 in the presence of stimulus 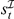 as a standard probabilistic function of i) the reinforcement expectations 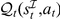 associated with that pair on that trial, and ii) a time-invariant, fixed, response bias 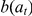:
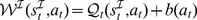
(1)

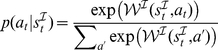
(2)where 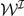 is the instrumental weight of action 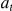, and where the variable 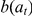 can take on value 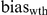 for withdrawal go actions, or 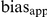 for the approach go actions. It is always zero for the nogo action. There was no delayed outcome in the instrumental task, and the expectations were thus constructed by a Rescorla-Wagner-like rule with a fixed learning rate 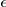. The immediate, intrinsic, value of the reinforcements delivered in the experiment may have different meaning for different subjects. To measure this effect, we added two further parameters: the reward sensitivity 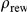 and the punishment sensitivity 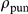, yielding an update equation for the expectations:
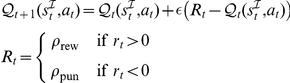
This is model 5 in Table 2, which has the lowest 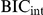 score (see below). Alternative models tested on the instrumental data only are as follows: Model 1 assumes that 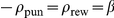, and that 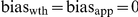. Model 2 allows only for separate reward and punishment sensitivities and model 4 for separate biases. Model 3 again assumes 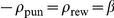, and that 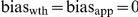, but allows for two separate learning rates, i.e. 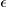 in Equation 3 is replaced by 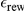 on trials where 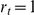, and by 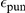 on trials where 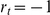. Model 6 and 7 are expansions of the final model, allowing for separate reward and punishment sensitivities (model 6) and for separate learning rates (model 7) in the approach and withdrawal conditions.

**Table 2:** Parameters contained in each of the models in Figure 3.

Model	Data			Parameters		Generalization	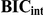
1	instrumental	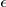	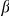				5000
2	instrumental	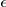	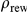 , 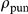				4613
3	instrumental	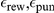	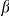				4665
4	instrumental	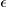	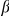	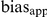 , 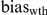			4771
5	instrumental	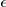	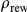 , 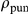	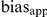 , 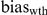			4606
6	instrumental	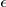	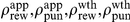	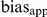 , 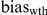			4618
7	instrumental	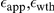	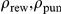	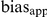 , 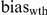			4626
8	instr&PIT	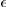	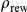 , 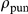	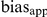 , 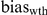	10 separate 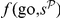	exact	17396
9	instr&PIT	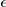	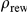 , 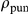	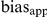 , 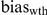	10 separate 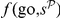	extinction	17634
10	instr&PIT	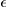	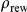 , 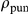	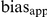 , 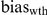	10 separate 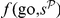	noisy	16769

Our main measure of interest is the effect of Pavlovian stimuli on the approach and withdrawal actions. Let additionally 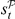 be the Pavlovian stimulus on trial 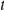. We can then write an equation similar to equation 2 for the trials where both instrumental and Pavlovian stimuli were present, but including a term 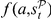 that quantifies the effect of the particular Pavlovian stimulus 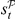 on the action 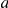. This means that the action weights due to the instrumental and Pavlovian controllers are added inside the exponent of equation 2, and that thus the probabilities each controller attaches to a particular action are multiplied and renormalized. The two controllers are therefore treated as two distinct entities, each separately voting for a particular action to be emitted. The influence of each system on action choice is *relative* to the strength with which the other enhances one particular action. We write the PIT weight of action 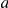 as:
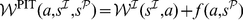
(3)Here we force 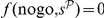 at all times. The go values 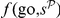 can take on 10 separate, inferred, values, meaning that there is one separate parameter for each of the five Pavlovian stimuli 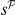 in each of the two blocks. Each of these parameters captures how much 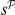 boosts the go over the nogo action (if 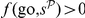) or the inverse (if 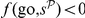). Note that because these are separately inferred, independent, parameters, this formulation does not impose any assumptions about the effect of the value of the stimulus 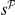, or about the relative effect of different stimuli 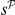 with different values. Hence, this controls for variation in learning during the Pavlovian training block (though the query trials indicate that learning was very robust).

Equation 3 (Model 8 in Table 2) assumes that the stimulus-action values 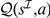 at the end of the instrumental block are perfectly and exactly generalized to the PIT block. We first tested an alternative model (Model 9 in Table 2) that included an exponential extinction factor, letting the 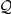 values decay on each PIT trial by 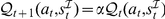 with 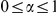. Next, we tested the model described in the main text (Model 10 in Table 2), which allowed for a fixed, Gaussian random offset between the effective 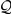 values in the instrumental and PIT stages, i.e. we wrote:
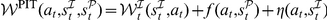
The noise factor 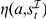 took on value 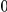 for the nogo action (akin to the bias and 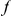 variables). It took on a separate value—which was inferred as a separate parameter—for each subject and each stimulus. However, all stimuli shared the same prior distribution for this noise variable. That is, in the E step of our EM procedure, we fitted one Gaussian mean and variance to all the 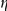
's that had been inferred for all stimuli for all subjects. In this sense, the generalization factors 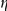 were drawn from one Gaussian prior whose mean and variance were fitted just like the mean and variance of the other parameters.

### Model fitting procedure

For each subject, each model specifies a vector of parameters 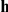. Assuming Gaussian prior distributions 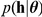, we find the maximum a posteriori estimate 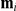 of the parameters for each subject 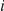:
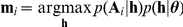
where 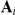 are all actions by the 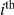 subject. We assume that actions are independent (given the stimuli, which we omit for notational clarity), and thus factorize over trials. The prior distribution on the parameters mainly serves to regularise the inference and prevent parameters that are not well-constrained from taking on extreme values. We set the parameters of the prior distribution 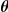 to the maximum likelihood given all the data by *all* the 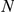 subjects:
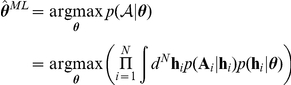
where 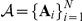. This maximisation is straightforwardly achieved by Expectation-Maximisation [109]. We use a Laplacian approximation for the E-step at the 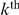 iteration:
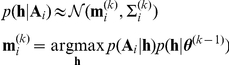
where 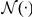 denotes a normal distribution over 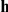 with mean 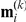 and 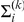 is the second moment around 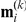, which approximates the variance, and thus the inverse of the certainty with which the parameter can be estimated. Finally, the hyperparameters 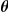 are estimated by setting the mean 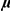 and the (factorized) variance 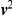 of the prior distribution to:
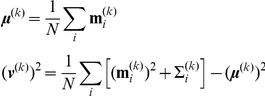
ansformed before inference to enforce constraints. Unconstrained parameters are inferred in their native space. These model fitting procedures were verified on surrogate data generated from a known decision process.

### Model comparison

We fitted a large number of different models to the data, and some of these models differ in their flexibility. For instance, Model 8, which assumes that the instrumental 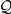 values are generalized exactly to the PIT stage is much less flexible than models 9–10, which allow for an offset. It is important to choose that model which is flexible enough to explain the data, but not so flexible that it would also fit very different data equally well [109].

Ideally, this is achieved by computing the posterior log likelihood 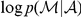 of each model 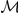 given all the data 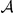. As we have no prior on the models themselves (testing only models we believe are equally likely a priori), we instead examine the model log likelihood 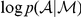 directly. This quantity can be approximated in two steps. First, the integral over 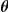 [110]:
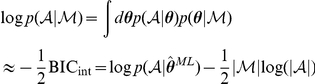
Importantly, however, 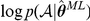 is not the sum of individual likelihoods, but in turn an integral over the parameters of each individual subject:
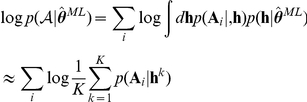
The second line shows that we approximated the integrals by (importance) sampling 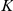 times from the empirical prior distribution 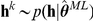 [109]. These samples were then also used to derive the error bars as the second moments around the maximum:
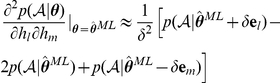
where 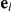 is a vector of zeros of the same dimension as 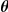 with only entry 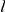 set to one. The shifted likelihoods can be easily computed by re-weighting the 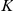 samples drawn before:
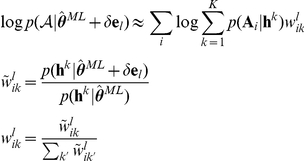
Note that while this model comparison procedure does give a good *comparative* measure of model fit, we still need an absolute measure to ensure that the best model does indeed provide a model fit that is adequate (even the best might be bad). Given each subject's MAP parameter estimate, we compute the total “predictive probability”:
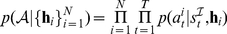
(4)where we suppressed the dependence on stimuli on the LHS for clarity. We note that 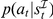 depends on the parameters 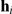, which have been fitted to the data. We term it a predictive probability in the sense that it predicts a subject's choice at time 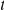 given that subject's *past* behaviour. We emphasize however, that this does depend on the MAP parameters 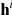 fitted to that subjects' entire choice dataset. Finally, we test whether the expected number of choices predicted correctly exceeds that expected by chance (using a binomial test). The overall predictive probability is given by the geometric mean over all choices and subjects: 
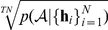
.

## Supporting Information

Text S1 Disentangling the roles of approach, activation and valence in instrumental and Pavlovian responding.
(PDF)
