# Supplementary material for: Disentangling the Roles of Approach, Activation and Valence in Instrumental and Pavlovian Responding
Source: PLoS Comput Biol. 2011 Apr 21;7(4):e1002028. doi: 10.1371/journal.pcbi.1002028 (PMC3080848; doi:10.1371/journal.pcbi.1002028)
Supplement: Text S1 — Disentangling the roles of approach, activation and valence in instrumental and Pavlovian responding. (PDF) [file pcbi.1002028.s001.pdf]

Disentangling the roles of approach, activation and valence in instrumental and Pavlovian responding

Quentin J.M. Huys[1,2,3,*], Roshan Cools[4], Martin Gölzer[5], Eva Friedel[5], Andreas Heinz[5], Raymond J. Dolan[1], Peter Dayan[2]
1 Wellcome Trust Centre for Neuroimaging, UCL, London, UK
2 Gatsby Computational Neuroscience Unit, UCL, London, UK
3 Medical School, UCL, London, UK
4 Donders Institute for Brain, Cognition and Behaviour, Centre for Cognitive Neuroimaging, Radboud University Nijmegen, Nijmegen, Netherlands
5 Charité Universitätsmedizin Berlin, Campus Charité Mitte, Berlin, Germany
∗ E-mail: qhuys@cantab.net

Supplementary Text S1

We tested two further explanations for the punishment insensitivity seen both in the raw and model data.

First, we asked whether it might be due to a choice stickiness [1], i.e. a tendency for subjects to stick to a particular choice independently of the task structure. Like the winning model 5 (in figure 3 of the main text), this model contained two separate reward and punishment sensitivities, one learning rate, and two biases for the approach and punishment condition. Stickiness was modelled by an additional, unconstrained, parameter γ, making the overall action weight:

$$\begin{aligned} \mathcal{W}^{\mathcal{I}}(s_t^{\mathcal{I}}, a_t) &= \mathcal{Q}_t(s_t^{\mathcal{I}}, a_t) + b(a_t) + g(a_t) \\ g(a_t) &= \begin{cases} \gamma & \text{if } a_t = a_{t-1} \\ 0 & \text{if } a_t \neq a_{t-1} \end{cases} \end{aligned}$$

Figure S1 shows the resulting parameters inference (fit using the same procedures as for the other models). On average, there is no stickiness (γ is not significantly different from zero Figure S1C). In addition, inclusion of this parameter does not affect the qualitative pattern of the other parameters: reward sensitivity ρ_{rew} is still much larger than punishment sensitivity (Figure S1A); and a bias against go is still selectively present in the withdrawal block (Figure S1B, bias_{wth}).

Second, we asked whether the punishment insensitivity might be due to emerging maximisation behaviour. While initially, the optimal behaviour often involves random foraging choices, after a while it becomes more advantageous to focus on the apparently optimal choice without further exploration. Intuitively, this might lead to an apparent insensitivity to punishments. We generated surrogate choice data in which the agent exploited progressively more. The data was generated from a $\mathcal{Q}$ learner with equal reward and punishment sensitivities. Prior to choice, the $\mathcal{Q}$ value was multiplied by a factor β that increased linearly in time—a standard approach to exploitation [2]. To this surrogate data we then fit a model with two separate reinforcement sensitivities (but effectively with fixed exploration parameter $\beta = 1$). However, in this model reinforcement sensitivities were additionally allowed to change over time. Figure S2A shows that this would predict equal, large, and increasing, reward and punishment sensitivities. Applying this time-varying fit to the experimental data reveals that, unlike what is predicted from exploitation, our subjects showed a progressive insensitivity to punishments (Figure S2B). Notably their sensitivity to punishments was less than that to rewards from the very beginning, and then rapidly declined further.

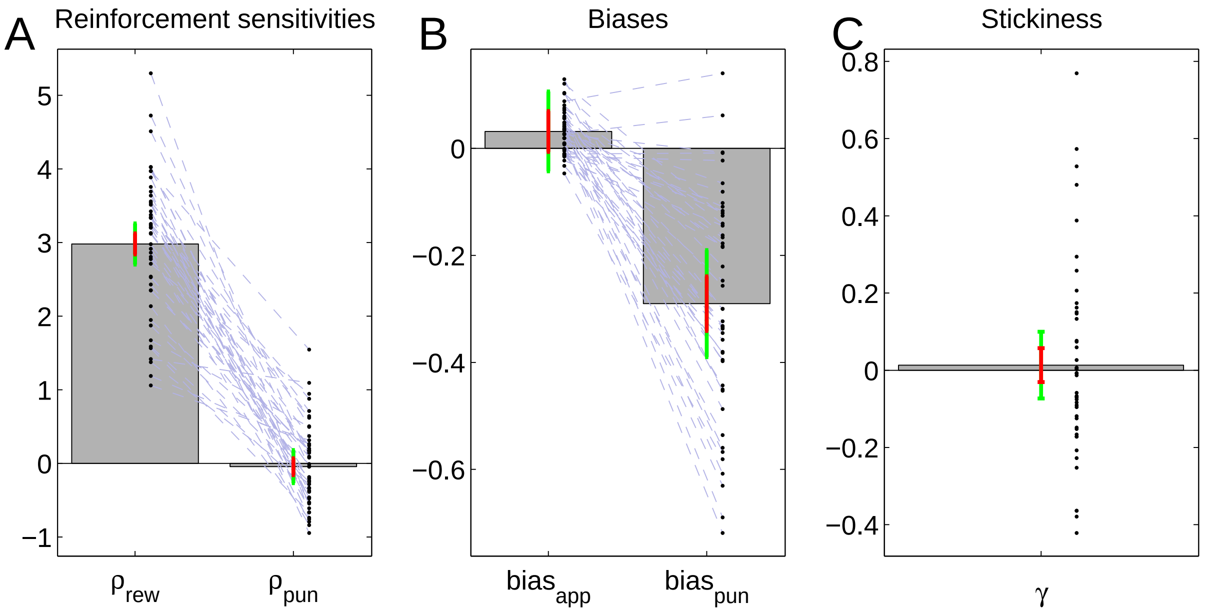

Figure S1. Parameters inferred when including a 'sticky choice'. **A**: Reinforcement parameters; **B**: Bias parameters; **C**: Sticky parameter γ. On average, the sticky parameter does not differ from zero. Inclusion of the sticky parameter does not alter the pattern of results. Critically, choice stickiness does not account for punishment insensitivity (which is not different from zero, $p > .5$ two-tailed t-test). Bars show the group means, red and green errorbars estimated standard errors and 95% confidence intervals. Black dots are individual data. Data from each individual are linked up by blue dashed lines.

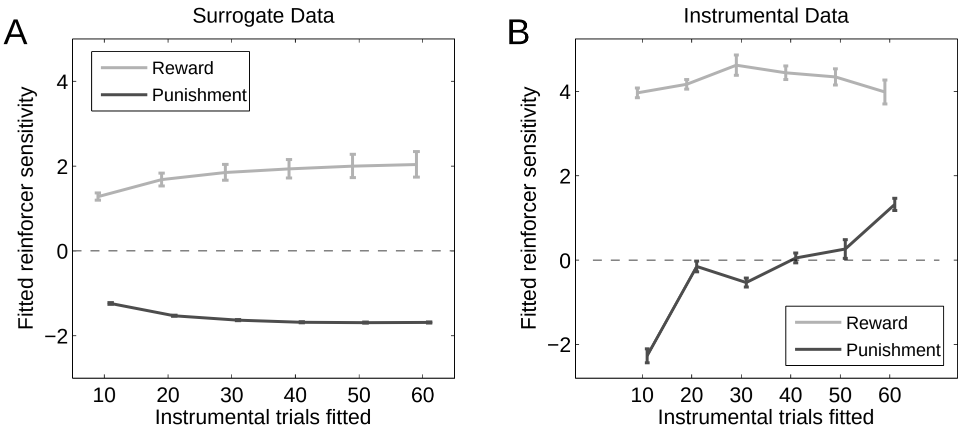

Figure S2. **A**: Surrogate data generated from the model with an overall reinforcement sensitivity β that increased linearly over time. As a deterministic strategy emerges (with a large β), inferred ρ_{rew} and ρ_{pun} become more accentuated with time. **B**: Time-varying reward and punishment sensitivity estimates. Neither of the two becomes more accentuated, as would be expected from an emerging deterministic strategy; and the effect of punishments vanishes rapidly.

References

1. Lau B, Glimcher PW (2005) Dynamic response-by-response models of matching behavior in rhesus monkeys. J Exp Anal Behav 84: 555–579.

2. Sutton RS, Barto AG (1998) Reinforcement Learning: An Introduction. Cambridge, MA: MIT Press. URL `http://www.cs.ualberta.ca/ sutton/book/the-book.html`.
